# Self-harm behaviour and externally-directed aggression in psychiatric outpatients: a multicentre, prospective study (viormed-2 study)

**DOI:** 10.1038/s41598-019-53993-7

**Published:** 2019-11-28

**Authors:** Paolo Scocco, Ambra Macis, Clarissa Ferrari, Mattia Bava, Giorgio Bianconi, Viola Bulgari, Valentina Candini, Giuseppe Carrà, Cesare Cavalera, Massimo Clerici, Giovanni Conte, Marta Cricelli, Maria Teresa Ferla, Laura Iozzino, Alberto Stefana, Giovanni de Girolamo

**Affiliations:** 1 Department of Mental Health, ULSS 6 Euganea, Padova, Italy; 2 Service of Statistics, IRCCS Istituto Centro San Giovanni di Dio Fatebenefratelli, Brescia, Italy; 3 Department of Medicine and Surgery, University of Milano Bicocca, Monza, Italy; 4 Department of Mental Health, ASST Ovest Milanese, Milano, Italy; 5 Unit of Epidemiological and Evaluation Psychiatry, IRCCS Istituto Centro San Giovanni di Dio Fatebenefratelli, Brescia, Italy; 6 Division of Psychiatry, University College London, London, UK; 7 Department of Psychology, Catholic University of the Sacred Heart, Milano, Italy; 8 Department of Mental Health, ASST of Monza, Monza, Italy; 9 Department of Mental Health, ASST Spedali Civili of Brescia, Brescia, Italy; 10 Department of Mental Health, Asst-Rhodense G.Salvini of Garbagnate, Milano, Italy

**Keywords:** Human behaviour, Schizophrenia

## Abstract

The aim of the project was to investigate differences between outpatients with Severe Mental Disorders (SMDs) with and without a history of Self-Harm behaviour (SHb) and/or Violent behaviour against other people (Vb) in relation to: (a) socio-demographic and clinical characteristics, (b) violent behaviour during a 1-year FU, (c) predictors of SHb and Vb during the FU. Outpatients with SMDs, with and without a history of Vb were enrolled. They were divided in four groups: patients with lifetime Vb (V), patients with both Vb and SHb (V-SH), patients with only SHb (SH) and patients with no history of SHb and Vb (control group, CONT). The frequency and severity of SHb and Vb during the FU were assessed every two weeks by the MOAS. Overall 246 patients were enrolled. BPRS-E Depression item, the SLOF Social acceptability, the BDHI Indirect Aggression, the BIS Motor Impulsiveness and the STAXI-2 Control-Out showed significant correlations with all the four groups (p < 0.030). V and V-SH patient groups reached higher scores in all MOAS sub-scales. Age among the SH group and BPRS-E affect-anxiety subscale among the V group significantly predicted aggression against people. In people with SMDs a history of SHb or Vb is associated with different medium-term outcomes.

## Significant outcomes

- Outpatients with a lifetime history of Vb and SHb showed more severe psychopathological symptoms compared to those with only a history of SHb or Vb or no such a history.
- A history of violence against others and self-harm, or only history of violence partially predicted future aggressive behaviour at 1-year FU. Ninety percent of controls and 82% of SH did not show any aggressive behaviour during the FU period, whereas 40% of Vb and SH patients aggressively behaved at least once. Of these, 13% showed both externally-directed aggression and SHb.
- In the control group, only a history of lifetime substance use and non-planning impulsivity did predict future externally-directed aggression during the FU.

## Limitations

- History of SHb was obtained from clinical records and was not based on a specific investigation.
- The medium duration of the FU (1 year) limited the possibility to identify a higher number of episodes of aggressive or violent behaviour, in particular of SH.

## Introduction

Violent and deliberate Self-Harm behaviour (SHb) of people with severe mental disorders (SMDs) represents a public health concern and a challenge for both clinicians and family members and can also have a profound impact on public opinion^1^. Deliberate SHb has been defined as an acute, non-fatal act carried out deliberately in the form of an acute episode of behaviour by an individual with variable motivations. The intention to end life may be absent or be present to a variable degree^2^.

A close correlation between ‘externally directed aggression’ (from now onwards ‘aggression’) and SHb has been hypothesized by various authors regardless of study methods and explanatory models (e.g., biopsychosocial, cognitive or interpersonal) used^3–10^.

People with a history of externally directed aggression are more prone to repeat similar acts against others^11^, or to engage in SHb^12^. Moreover, those who attempt suicide are at risk of repeating this behaviour^13,14^ or exhibiting other violent behaviour^2^. SHb and externally directed aggression share some risk factors^3^, including the presence of a SMD, substance use disorders^15–18^ and being exposed to trauma or neglect during childhood^19^. Other factors, such as explosiveness, irritability, anger, frustration and hostility, which characterize reactive aggression, are also associated with SHb^20,21^; more specifically, impulsivity seems to play a significant role in SHb^22^.

Anger has also been detected in SHb^23^ but it is still unclear how it intervenes in a predisposition to aggression and how it correlates with other variables, such as impulsivity and emotional dysregulation^24^.

Fazel and colleagues^25^ showed that risk factors for violent crimes, suicide, and premature mortality included both those specific to individuals with schizophrenia and related disorders and those shared with the general population.

Despite these contributions to the field, a clear identification of specific risk factors involved in the simultaneous presence of SHb and externally directed aggression remains an area of ongoing research.

## The Italian System of Mental Health Care

After the closing of all mental hospitals in Italy due to law 180, approved in 1978, a nation-wide network of 145 Departments of Mental Health (DMHs) now delivers outpatient and inpatient care, and also run residential facilities (the latter are 2,346 with 26,310 beds). According to the latest annual report on Italian mental health services^26^, in 2017 DMHs were caring for 851,189 people >18 years of age (1.67% of the general population >18 years of age). Hospital care is delivered through 318 small general hospital psychiatric units, with an average of 12.5 beds per unit. In terms of diagnostic profiles of patients in treatment, among the 169.4 patients per 10,000 general population in treatment there were 35.8 with a diagnosis of schizophrenia spectrum disorders, 39.2 with a diagnosis of unipolar depression, 14.3 with a diagnosis of bipolar disorder and 12.0 with a diagnosis of personality disorder. The Ministry report showed that there is marked quanti- and qualitative variation in the provision of out- and inpatient care throughout the country, and service utilization patterns are similarly uneven.

### Aims of the Study

The main aim of this study is to investigate the differences between outpatients with SMD who had a history of SHb and/or violent behaviour against other people (Vb) and patients who had no such history, in relation to: (a) sociodemographic and clinical characteristics at baseline; (b) evolution of the aggressive and violent behaviour during a 1-year follow-up (FU); and (c) predictors of SHb and Vb during a 1-year FU.

## Methods

### Study design

This study is part of the Violence Risk and Mental Disorder (VIORMED-2), a prospective cohort study. The study involved outpatients in treatment at different community mental health centres (CMHCs) belonging to four Departments of Mental Health (DMHs), all located in Northern Italy. In each of the four DMHs, there were four CMHCs providing outpatient care; the participating sites had a catchment area with an average population of 351,400 (±32,366.70). The average number of outpatients under care was 4,206 (±360.13).

Treating clinicians recruited outpatients during a 6-month period in 2015. Inclusion criteria were a primary psychiatric diagnosis and age between 18 and 65 years. Exclusion criteria were having a primary diagnosis of major neurocognitive disorders, intellectual disability or sensory deficits and/or having a primary substance use disorder. The selection of patients with a history of physical violence against others (cases) was based solely on a comprehensive and detailed documentation (as reported in clinical records) about a history of violent behaviour(s). They had to meet one of the following criteria: (i) to have been admitted at least once to a forensic mental health hospital (FMH) for any violent acts against people; (ii); (ii) to have a documented lifetime history of violent acts against people in the last 10 years (as reported in clinical records), which caused (or might have caused) physical harm to the victim, or having committed armed robbery, pyromania or sexual violence; this may have led to arrest. Control patients (without any history of violence against other people) were matched by age, sex and diagnosis.

Before signing consent, the treating clinician with the local research assistant provided the potential participant with detailed information about the observational nature of the study, of the study aims and methods. The participant information sheets and consent/assent forms made explicit the voluntary nature of subjects’ involvement and the possibility to withdraw from the study at any time. Among outpatients, there were 6 patients who had a legal representative: 3 ‘cases’ and 1 control in Garbagnate; 1 control in Legnano and 1 case in Brescia. In these six cases the informed consent was initially sought from the legal representative, and then from the patient. Even if the legal representative gave consent but the patient refused, that person was not included in the study. Ethical approval was granted by the ethical committee of the coordinating center (IRCCS Saint John of God, Fatebenefratelli; n° 64/2014) and by ethical committees of all other recruiting centers (EC of the Monza Health Unit, letter dated December 21^st^, 2015, approved on November 26^th^, 2015; EC of the Garbagnate Health Unit, approved on July 7^th^, 2015, n. 00397/2015/DG; EC of the Legnano Health Unit, approved on July 14^th^, 2015, n. 220/15; EC of the Brescia Health Unit, approved on April 10^th^, 2015, n. NP 1952) (as described in our previous publications on the project, see Barlati *et al*.^27^). As agreed with the ECs, all methods were performed in accordance with the relevant guidelines and regulations.

### Measures

A specific patient schedule was developed to collect information on selected sociodemographic characteristics and clinical and treatment-related factors^27^.

Symptom severity and psychosocial functioning were assessed using the Brief Psychiatric Rating Scale-Expanded (BPRS-E)^28^ and the Specific Levels Of Functioning (SLOF)^29^.

Violence and impulsivity were evaluated using the following instruments: (a) the Brown-Goodwin Lifetime History of Aggression (BGLHA), an 11-item questionnaire^30^ assessing lifetime aggressive behaviour across two stages of life (adolescence and adulthood); (b) the Buss-Durkee Hostility Inventory (BDHI), a 75-item questionnaire developed to assess eight subscales related to hostility and negative affect30; (c) the Barratt Impulsiveness Scale (BIS-11), a 30-item, 4-point Likert scale questionnaire that investigates personality and behavioural impulsiveness, resulting in scores ranging from 30 to 120^31^; (d) the State-Trait Anger Expression Inventory 2 (STAXI-2), which includes six scales plus an Anger Expression Index, an overall measure of total anger expression^32^ (please see also previous publications on this project, de Girolamo *et al*., 2016^33^).

The Millon Clinical Multiaxial Inventory – III (MCMI-III) was used to assess patients’ personality profiles. It is composed of 175 true-false questions, including 14 personality scales, 10 clinical syndrome scales and four correction scales^34^. A score >84 indicates that the patient displays all the symptoms at a diagnostic level, so it is possible to speak of a full-blown PD; scores 75–84 suggest the presence of maladaptive traits and subthreshold symptoms (not at a diagnostic level). Finally, scores <75 are generally considered not clinically relevant^35^.

### Assessment of self-harm

SH history was based on all information contained in the official clinical records and was reported in the patient schedule.

For the purposes of this study, patients were divided into four groups: (a) patients with a history of violent behaviour against other people (but not SHb) (V); (b) patients with a history of both Vb and SHb (V-SH); (c) patients with a history of SHb (but not Vb) (SH); and (d) patients with no history of violent behaviour against other people and of SHb, named controls (CONT).

All instruments were administered by clinical psychologists who received specific training for each instrument and were given direct supervision throughout all stages of the study. Baseline assessment included five or six sessions, for an average of 8 hours of baseline assessment for each patient. From the first session, baseline assessments for each patient were completed within one month.

### Longitudinal monitoring of aggression and self-harm

The FU period started as soon as patients had completed baseline assessments. Aggressive or violent behaviour and SHb exhibited by patients during the 1-year FU were rated every fifteen days with the Modified Overt Aggression Scale (MOAS)^36^, for a total of 24 MOAS ratings for each patient. The MOAS includes four aggression subdomains: verbal, against objects, self-aggression, and against people l. A score from 0 to 4 is assigned: 0 indicating no aggressive behaviour and higher scores showing increasing severity. The score is then weighted with a value assigned to each category: 1 for verbal aggression, 2 for aggression against objects, 3 for self-aggression and 4 for aggression against people. The total weighted score for each evaluation ranges from 0 (no aggression) to 40 (maximum grade of aggression); since there were 24 ratings during a 1-year period, the individual MOAS total score for that time period ranged from 0 to 960 (Barlati *et al*., 2019^27^).

### Statistical analyses

Summary statistics were carried out through frequencies and percentages for discrete variables and by means and standard deviations (SDs) for continuous variables. To compare categorical data, the χ2 test or exact Fisher’s test, when appropriate (n < 5 in any cell), were used. Normality assumption was verified through the visual inspection of QQ-plots and box plots in addition to Shapiro-Wilk and Kolmogorov-Smirnov tests.

For quantitative data, the ANOVA or non-parametric Mann Whitney test were used (on the basis of the variables’ distribution). Post-hoc analyses were computed by Bonferroni correction.

A multinomial logistic model was adopted to evaluate which variables were more associated with the groups using a backward stepwise procedure to obtain the final best model in terms of goodness of fit. Stuart-Maxwell test was performed to compare the distribution of aggressive behaviours over time among the four groups.

Finally, given the non-Gaussian distribution of MOAS scores (skewed and zero-inflated distribution), the analysis of predictive factors of violence was performed by adopting generalized linear models (GLMs) with tweedie distribution and log-link function (MOAS scores, total and subscales, used as dependent variables and all the other measures as independent ones). The beta coefficients are reported as exponential reparameterization of the standardized ones to make them easier to interpret.

All tests were two-tailed with the statistically significance level set at p = 0.05. All data were coded and analysed using the Statistical Package for Social Science^37^, and R: A language and environment for statistical computing^38^.

## Results

### Sample characteristics

The overall sample included 246 patients: 29.7% (N = 73) of them belonged to V group, 21.5% (N = 53) were part of V-SH group, 13.8% (N = 34) were in the SH group and the remaining 35% (N = 86) were controls. Among patients who had a history of SHb (in both V-SH and SH groups), 7 patients attempted suicide in the 12 months prior to the baseline assessment.

No significant differences among groups were found for age, nationality, marital status, education and occupation (Table 1S). The four groups differed by gender (p = 0.014) showing a dominant percentage of females in the SH group. Moreover, it emerged that patients in the V-SH and V groups spent significantly more time doing nothing (p = 0.036).

**Table 1:** Clinical characteristics of the sample (N = 246).

	V N=73 n (%)	V-SH N=53 n (%)	SH N=34 n (%)	CONT N=86 n (%)	*p-value*
**Primary diagnosis as established by treating clinicians**					
Schizophrenia	33 (45.2)	19 (35.8)	8 (23.5)	43 (50.0)	0.083
Personality disorder	24 (32.9)	23 (43.4)	14 (41.2)	20 (23.3)	
Bipolar disorder	10 (13.7)	3 (5.7)	5 (14.7)	9 (10.5)	
Anxiety/Mood disorders	6 (8.2)	8 (15.1)	7 (20.6)	14 (16.3)	
**Comorbidity with substance/alcohol abuse (Secondary diagnosis)**					
Alcohol	4 (5.5)	5 (9.6)	3 (8.8)	1 (1.2)	**0.014**
Other substances	5 (6.8)	8 (15.4)	9 (26.5)	8 (9.3)	
None	64 (87.7)	39 (75.0)	22 (64.7)	77 (89.5)	
**In pharmacological treatment**					
Yes	66 (90.4)	49 (94.2)	33 (97.1)	81 (94.2)	0.640
No	7 (9.6)	3 (5.8)	1 (2.9)	5 (5.8)	
**Lifetime Alcohol use**					
Yes	20 (27.4)	19 (35.8)	11 (33.3)	16 (18.6)	0.118
No/Occasional	53 (72.6)	34 (64.2)	32 (66.7)	70 (81.4)	
**Lifetime Substances use**					
Yes	23 (31.5)	24 (47.1)	10 (30.3)	15 (17.4)	**0.003**
No	50 (68.5)	27 (52.9)	23 (69.7)	71 (82.6)	

V: patients with a history of violent behaviour against other people (but not self-harm behaviour); V-SH: patients with both a history of violent behaviour against other people and self-harm behaviour; SH: patients with a history of self-harm behaviour; CONT: patients with no history of violent behaviour against other people and of self-harm behaviour.

p value was obtained through χ^2^ test or Fisher’s exact test (when n < 5 at least in one cell).

There was a significantly higher proportion of patients in the V-SH group (Table 1S). who were witnesses or victims of episodes of physical violence in the family.

No differences were found among the four groups for the primary diagnosis and for the prescription of psychotropic medications. Differences were instead observed for a comorbidity with substances/alcohol abuse (p = 0.014) and lifetime substance use (p = 0.003), but not for lifetime alcohol use (Table 1).

### History of Violence

There were differences among groups in terms of lifetime history of aggression and violent behaviour across adolescence and adulthood as assessed by the BGLHA (p < 0.001), showing that V-SH scores were significantly higher than CONT ones (Table 2S).

**Table 2:** Clinical variables associated with the four groups **(multinominal logistic model)**.

Variable	p-value	OR (p-value)
**BPRS-E Depression**	**0**.**013**	
V vs V-SH		0.47 (0.014)
V vs SH		0.41 (0.009)
**BDHI Indirect Aggresssion**	**0**.**006**	
V vs V-SH		0.63 (0.004)
V vs SH		0.63 (0.019)
**SLOF Social Acceptability/Adjustment**	<**0**.**001**	
V vs SH		0.61 (<0.001)
V vs CONT		0.67 (<0.001)
V-SH vs SH		0.59 (<0.001)
V-SH vs CONT		0.65 (<0.001)
**BIS Motor Impulsiveness**	**0**.**030**	
V vs CONT		1.15 (0.029)
SH vs CONT		1.18 (0.027)
**STAXI-2 Anger control-out**	**0**.**002**	
V vs CONT		0.95 (0.016)
V-SH vs CONT		0.93 (0.004)
SH vs CONT		0.95 (0.035)

BPRS-E: Brief Psychiatric Rating Scale-Expanded; BDHI: Buss-Durkee Hostility Inventory; SLOF: Specific Levels Of Functioning; BIS: Barratt Impulsiveness Scale; STAXI-2: State-Trait Anger Expression Inventory 2.

V: patients with a history of violent behaviour against other people (but not self-harm behaviour); V-SH: patients with both a history of violent behaviour against other people and self-harm behaviour; SH: patients with a history of self-harm behaviour; CONT: patients with no history of violent behaviour against other people and of self-harm behaviour.

OR = Odds Ratio, representing the probability to belong to the first group with respect to the second.

### Psychopathology

Considering the four-factor of the BPRS-E^26^, V and V-SH patients showed significantly higher scores (and therefore greater symptom severity) than CONT in ‘Activation’ factor (p = 0.002 and p = 0.038 respectively). Moreover, significant differences (p = 0.040) among patients were found in terms of depression (assessed by item 3 of BPRS-E) showing that the V-SH group reported significantly higher scores than the V group (Table 2S).

### Psychosocial Functioning

The only significant difference in SLOF ratings has been found for the ‘Social acceptability/adjustment’ subscale (p < 0.001). Post-hoc tests show that V-SH patients had lower scores than all the other three groups and that V patients had lower scores than SH and CONT (Table 2S).

### Hostility, Impulsivity and Anger

Other differences were found for assault (p = 0.046) and indirect aggression (p < 0.001), as assessed by the corresponding BHDI subscales (Table 2S): V-SH group had significantly higher scores of BDHI ‘Assault’ than V group and patients with a history of SHb (V-SH and SH) had higher scores of BDHI ‘Indirect Aggression’ than patients with no SHb (V and CONT).

Groups differed also in terms of BIS ‘Motor Impulsiveness’ (p = 0.028) and of BIS total score (p = 0.035). Finally, regarding anger (STAXI-2), there were statistically significant differences only for the ‘Anger control-out’ and for the Anger Expression Index subscales (p = 0.001 and p = 0.020 respectively). In detail, V-SH patients were less able to control the anger (*p* < 0.001) and had a higher “Anger expression Index” (*p* = 0.027) compared to controls (Table 2S).

### Personality disorders

No differences were found in terms of presence of personality traits or disorders assessed by the MCMI-III (Table 3S).

**Table 3:** Predictors of MOAS Aggression-against-People score: generalized linear models (GLMs) with interaction effect between variables and groups (all sample), and corresponding GLMs for the four groups.

Independent variables	Dependent variable = MOAS Aggression against People				
	All sample	Groups			
		V	V-SH	SH	CONT
	p-value	β_V_	β_V-SH_	β_SH_	β_CONT_
**Lifetime Substances use** (Yes *vs* No)	0.008	1.35	0.74	20.70	38.55**
**BIS** Unplanned impulsiveness	0.015	0.96	1.08	1.14	0.68*
**Age**	0.039	0.96	0.99	0.83**	0.89
**BPRS Affect-Anxiety**	0.043	0.84*	1.13	0.93	1.20

V: patients with a history of violent behaviour against other people (but not self-harm behaviour); V-SH: patients with both a history of violent behaviour against other people and self-harm behaviour; SH: patients with a history of self-harm behaviour; CONT: patients with no history of violent behaviour against other people and of self-harm behaviour.

p-value: significance of the interaction term between the group variable and the independent variables

*p < 0.05; **p < 0.01

β: exponential transformation of the coefficients of the Generalized Linear Models.

Βv, β_V-SH_, β_SH,_ β_CONT_: estimates of the variable effect in the four groups separately.

### Features associated with the four study groups

To identify the features which were associated to the four groups, a multinomial logistic regression was performed. All variables which differed among groups on univariate analyses (results shown in Table 1, Table 1S, Table 2S, and Table 3S) were entered as independent variables, while the Group variable (with 4 levels: V, V-SH, SH, CONT) was the dependent one. At a first step, all the variables were entered in the model and then, those non-significant, were removed. The final model including only the variables significantly associated with the Group variable. The variables more strictly associated with the four groups were the BPRS-E ‘Depression’ item (p = 0.013), the SLOF ‘Social acceptability/adjustment’ (p < 0.001), the BDHI ‘Indirect Aggression’ (p = 0.006), the BIS ‘Motor Impulsiveness’ (p = 0.030) and the STAXI-2 ‘Anger Control-Out’ (p = 0.002) (Table 2).

In detail, an enhancement of one point of the BPRS-E depression score decreased the probability of belonging to the V group with respect to V-SH or SH ones respectively of 53% (OR = 0.47) and 59%, (OR = 0.41). Similarly, happens for the BDHI’Indirect Aggression’. An enhancement of one point of the SLOF’Social acceptability/adjustment’ score decreased the probability to belong to the V and V-SH groups than to SH (OR = 0.61, OR = 0.59 respectively) and CONT (OR = 0.67, OR = 0.65 respectively). Similar results were found for STAXI-2 “Anger Control-Out” subscale. Finally, the increase of the BIS “Motor-Impulsiveness” score was associated to an enhancement of the probability to belong to V and SH groups with respect to CONTs of 15% and of 18% respectively (OR = 1.15, OR = 1.18).

### Self-Harm and Aggressive Behaviour during the 1-Year FU

During the 1-year FU three patients (one in the SH group and two in the V-SH group) attempted suicide (with ascertained suicidal intention) and a SH patient committed suicide.

**Figure 1:**
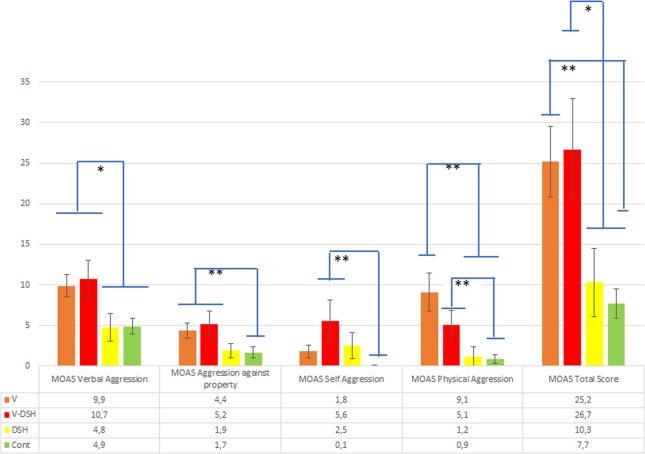
MOAS scores in the four study groups. V: patients with a history of violent behaviour against other people (but not self-harm behaviour); V-SH: patients with both a history of violent behaviour against other people and self-harm behaviour; SH: patients with a history of self-harm behaviour; CONT: patients with no history of violent behaviour against other people and of self-harm behaviour. *p < 0.05; **p < 0.01. The bars represent the post-hoc comparisons.

The four groups significantly differed on MOAS scores: verbal aggression (p = 0.001), aggression against objects (p < 0.001), self-aggression (p = 0.006), aggression against people (p < 0.001) and total score (p < 0.001) (Fig. 1), showing that V and V-SH had higher scores in all the subscales.

Considering our second aim (i.e., to assess how many patients maintained or changed their aggressive and violent behavior during the 1-year FU) the four groups (V, V-SH, SH and CONT) were redefined using MOAS scores in order to study the evolution of Vb and SHbover time. More in detail, we defined the groups using the MOAS ‘Self-aggression’ and the MOAS ‘Aggression against people’ subscales, coherently with the baseline definition (based on physical violence and on SHb). The four groups were therefore the following: (i) V patients with a MOAS score higher than zero in the ‘Aggression against people’ subscale and a MOAS ‘Self-Aggression’ score equal to zero; (ii) V-SH patients with both MOAS ‘Aggression against people’ and ‘Self-Aggression’ scores higher than zero; (iii) SH group with a MOAS ‘Aggression against people’ score equal to zero and a MOAS ‘Self-Aggression’ score higher than zero; (iv) CONT with MOAS ‘Aggression against people’ and ‘Self-Aggression’ scores equal to zero.

**Figure 2:**
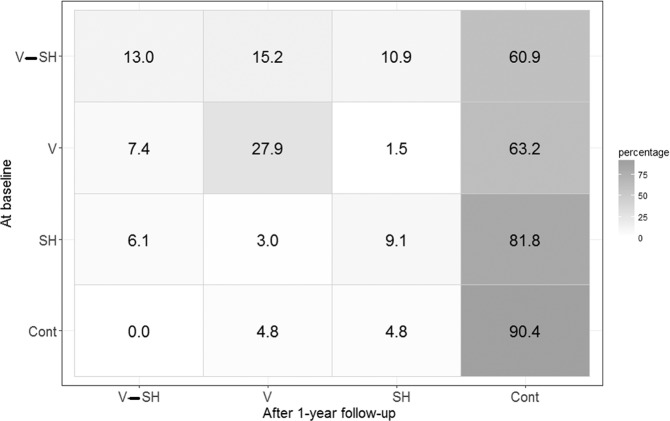
The evolution of aggressive and violent behaviour over time: percentage of patients in different groups at baseline and after the 1-year FU. (At baseline): V: patients with a history of violent behaviour against other people (but not self-harm behaviour); V-SH: patients with both a history of violent behaviour against other people and self-harm behaviour; SH: patients with a history of self-harm behaviour; CONT: patients with no history of violent behaviour against other people and of self-harm behaviour. (After 1-year FU): V: patients had a MOAS ‘Aggression against people’ score higher than zero and a MOAS ‘Self-Aggression’ score equal to zero; V-SH: patients with both MOAS ‘Aggression against people’ and ‘Self-Aggression’ scores higher than zero; SH: patients with a MOAS ‘Aggression against people’ score equal to zero and a MOAS ‘Self-Aggression’ score higher than zero; CONT: patients with a MOAS ‘Aggression against people’ and ‘Self-Aggression’ scores equal to zero.

The analysis was performed considering 230 patients for whom MOAS scores were available. Figure 2 (representing the confusion matrix) shows the percentage of patients that moved from the baseline groups (rows) to the FU ones (columns). The results showed that the most ‘stable’ patients belonged to the controls (90.4% of these patients continued to show the same behavioral patterns reported in their history). A moderate ‘stability’ characterized the V group (27.9% of patients remained in that the same group after 1-year FU). For what concerns the V, V-SH and SH groups, the majority moved to the CONT group (they did not show externally-directed aggression or SHb during the one-year FU), while some patients (belonging to any groups) changed their violent behaviours. The Stuart Maxwell test showed that the two patients’ distributions (the baseline and at the FU one) were significantly different (p < 0.001).

### Factors predicting aggressive behaviour

Finally, we assessed which variables had different effects (in the four groups) on the aggressive or violent behaviour (evaluated by MOAS). For this purpose, different GLMs (one for each predictor) have been performed using the MOAS scores (total and subscales) as dependent variable and potential predictors (all socio-demographic and clinical variables), the group variable and group*predictors interaction as independent ones. In particular, we focused on the MOAS subscales of ‘self-aggression’ and ‘aggression against people’ since these were the two dimensions of violence strictly related with the four groups.

For the MOAS ‘self-aggression’ subscale, no significant interactions between the analysed variables and the group were found, meaning that all the factors associated with the ‘self-aggression’ subscale had the same effect in the all four groups. Conversely, some factors were associated with the MOAS ‘aggression against people’ subscale in a different way among the 4 groups (Table 3). In detail, in the CONT group a lifetime substance use significantly increased the MOAS scores, while a unit increase in the BIS ‘Unplanned impulsiveness’ score was significantly associated with a mean decrease of 32% (β = 0.68) of MOAS ‘aggression against people’ rating. This inverse relationship is mainly due to a small percentage (4.8%) of controls, with low mean score in BIS ‘Unplanned Impulsiveness’ (21.7) and with substances abuse, who showed aggressive behaviours during the 1-year FU (with a MOAS Aggression against people higher than zero). Moreover, when age increases of a unit, the MOAS subscale score decreases in mean of 17% (β = 0.83) in the SH group. Finally, a unit increases of BPRS ‘Affect-Anxiety’ score in the V group was associated to a decrease of 16% (β = 0.84), on average, of the MOAS aggression against people subscale.

Data on predictors of MOAS ‘total score’, ‘verbal aggression’ and ‘aggression against objects’ are available on supplementary Table 4S.

## Discussion

To our knowledge, this is the first study that used a longitudinal design to evaluate externally-directed aggression and SHb in a sample of outpatients with SMDs.

Patients with a history of both violence against others and SHb (V-SH) displayed more severe clinical features and exhibited significantly higher scores in many psychopathological dimensions (e.g., hostility, direct and indirect aggression, motor impulsivity, anger), in the BPRS-E activation factor (which includes dimensions such as hostility, blunted affect, grandiosity, suspiciousness, lack of cooperation, excitement, hyperactivity) and exhibited more frequently a lifetime substance use, a worse functioning in daily life (i.e., they spent more than three hours per day doing nothing) and lower acceptability and social adaptation compared to controls.

Higher levels of motor impulsivity and anger in patients with both externally directed aggression and SHb had already been reported in the literature^4,23^.

Although subjects with a history of SHb (with or without violent behaviour against others) were more frequently diagnosed with a personality disorder (more than 40%), personality profile evaluated by MCMI-III did not show any differences among groups.

However, all these differences shown above were less visible comparing the other two groups (V and SH) and controls.

As expected, the logistic model showed that V, V-SH but also SH patients displayed a lower ‘externally directed aggression anger control’ score, while V and SH displayed higher ‘motor impulsivity’ score compared to controls. The only significant difference between the SH and V-SH groups was related to the greater social adaptation and acceptability of the former over the latter.

Interestingly, the intensity of indirect aggression significantly distinguished the three groups of aggressive patients: in particular, V patients presented lower levels of ‘indirect aggression’ than both V-SH and controls.

Daukantaite and colleagues^39^ suggested that SH does not serve to regulate negative affect in general, but is rather a specific subcategory of negative affect that centrally involves aggressive feelings, towards both others and towards self. These authors found that in their sample of young SH patients, the levels of direct and indirect forms of aggression and victimization were similar.

As already widely reported in literature^40,41^, SH patients reported a high prevalence of depressive and anxiety disorders (20.6%). However, in terms of dimensional assessment (as done by the BPRS depression subscale), patients in the V-SH group were significantly more depressed than V. A diagnosis of depression has been associated with an increased risk of violence^42^, and more severe depression has been associated with more serious and frequent violent behaviours in patients with schizophrenia^43^. However, our sample was represented by patients who suffered from various mental disorders, not just schizophrenia and depression.

By logistical analysis we have seen that low anger control was a common factor in all the groups with aggression problems. Research on the relationship between anger, expression of anger (expressed as SHb or external aggression) and suicidal behaviour has led to equivocal results. Cross-sectional studies seem to indicate that suicidal young people have higher levels of trait anger than their non-suicidal peers. However, this feature is more common among those with a history of suicidal attempts, although it does not differentiate between those with different histories of suicidal behaviour. Furthermore, the relationship with suicidality disappears by controlling for covariates^44^.

Moreover, V and SH groups both showed higher scores in motor impulsiveness and lower scores in externally directed aggression anger control, and they have very different scores in terms of social acceptability. Indeed, lack of self-control and motor impulsiveness are two characteristics of impulsiveness which play an important role in the emergence of aggression^45,46^ and might be related to the ‘affective aggression’ (reactive, fear-induced, maternal, aggression)^47^.

### Are outpatients with a history of violence and/or SHb more likely to commit similar acts?

During the FU, four patients attempted suicide (with ascertained suicide intent): all of them had a history of at least one episode of SHb. This low recurrence rate is explained either by the relatively low rates of suicide attempts in Italy^48^, and/or as a result of the protective role of the FU procedure due to the study setting (e.g., the knowledge of being monitored as part of a study, the frequency of appointments, etc.)^49^. Although the monitoring with the MOAS was done asking key informants (e.g., clinicians, staff, family members) about the patient’s behaviour, the patient knew that a monitoring was in place and this may have somehow changed his behaviour, reducing his propension to self-harm.

V-SH patients showed more violent behaviour compared to both controls and SH patients, but no differences were found between the V-SH and V groups.

The prevalence of externally directed aggression and SHb during the 1-year FU in the four groups was significantly different from the history collected at baseline. However, the rate of co-occurrence defined by the presence of violence or SHb was relatively low, 26.5%. Almost all participants among controls and 82% of SH patients did not show any aggressive behaviour during the FU monitoring. Conversely, 40% of V-SH patients showed at least one aggressive behaviour (externally directed or self-directed), although only 13% of the sample showed both violent externally directed aggression and SHb.

The history of both past violence and/or SHb can provide a reliable prevision of the risk of future violent behaviour for only a specific part of our sample. The following question still needs an answer: ‘*Why do some subjects who display aggressive behaviour not engage in SHb or vice versa?’*.

Controls with a positive history of substance use had significant higher probability of exhibiting aggressive behaviour during the FU. This is consistent with previous studies^17,25^ and highlights the risk that substance use is one of the principal dynamics (or modifiable) risk factors for violence in psychosis. Contrary to previous data^17^ among controls, a higher unplanned impulsiveness level was associated with a lower risk of aggressive behaviour. This inverse relationship is mainly due to a large percentage (about 95%) of controls who did not exhibit aggressive behaviours during the 1-year FU and had high BIS unplanned impulsiveness score. This might be explained by the fact that impulsivity may be a mediator of violent behaviour when anger is also present^50^, and may also be involved in SHb^51^.

However, Vitiello *et al*.^46^ pointed out that in patients who manifested a predatory aggression (planned and goal-oriented), it was more frequent to history of drug abuse.

### Study limitations

This study has some limitations that are worth emphasizing. First of all, the design of the VIORMED study did not include a SHb classification and these behaviours were extracted from patients’ clinical charts. However, the detailed medical history and the numerous tests used have allowed us to select SH patients with good reliability. The lack of information about the presence or absence of suicidal ideation and planning in patients with SHb did not allow us to distinguish them in attempted suicide or SH. This did not allow a distinction between patients who intended to die and those who had no suicidal intent (thus distinguishing SH from attempted suicide)^15^.

The 1-year FU monitoring period reduced the possibility to detect new episodes of aggressiveness known to be at relative low frequency, in particular SHb; a longer follow-up period would have been desirable, but funding limitations prevented us from an extension of the follow-up.

The MOAS were completed taking into account all available information, but it was not based on continuous observation of the patient 24 hours a day. Thus, our results may have underestimated the occurrence of aggressive behaviours. Furthermore, no systematic assessment of symptomatology (and changes in it, if any) was conducted during or after the FU, which does not allow for an analysis of the relationships between such clinical variables and aggressive behaviour.

Although preponderance of female subjects might be expected in SH group^52,53^, the VIORMED study design (with controls paired with cases in terms of age, gender and diagnosis) may have affected the gender distribution of the sample.

### Implications

The current study offers new insights into the association between psychopathology, violence and SHb. Our results show that a previous lifetime history of externally directed aggression and SHb is an important predictor of new aggression among psychiatric outpatients. These findings suggest that SH harm in combination with aggression are warning signs of severe psychopathology that require advanced and specialised help.

Therefore, it is crucial to provide a specific anamnestic assessment to detect patients’ personal history of violence and SHb to recognize the co-occurrence of both forms of aggressiveness. However, the lack of externally-directed aggression and SHb in the patient’s history does not exclude their future onset in the presence of comorbid substance abuse and low levels of non-planning impulsiveness: this suggests that risk assessment and management might have to be carefully tailored to specific individuals.

The results of our study somewhat support that structured risk assessment can help a prediction of aggression and SHb, but their validity is modest and does not support a sole reliance on such tools for the management of individuals with SMDs with risk of SH or externally-directed aggression.

Moreover, our findings may assist clinicians in identifying appropriate indicators for violence and SHb prevention and promote more appropriate treatment programmes. This study might also allow researchers to design further studies taking into account both externally directed and self-harm behaviours.

## Supplementary information

Supplementary information

## Data Availability

The data that support the findings of this study are available from the corresponding author upon request.
