# Publisher Correction: Self-harm behaviour and externally-directed aggression in psychiatric outpatients: a multicentre, prospective study (viormed-2 study)

**DOI:** 10.1038/s41598-019-56963-1

**Published:** 2019-12-31

**Authors:** Paolo Scocco, Ambra Macis, Clarissa Ferrari, Mattia Bava, Giorgio Bianconi, Viola Bulgari, Valentina Candini, Giuseppe Carrà, Cesare Cavalera, Massimo Clerici, Giovanni Conte, Marta Cricelli, Maria Teresa Ferla, Laura Iozzino, Alberto Stefana, Giovanni de Girolamo

**Affiliations:** 1 Department of Mental Health, ULSS 6 Euganea, Padova, Italy; 2 Service of Statistics, IRCCS Istituto Centro San Giovanni di Dio Fatebenefratelli, Brescia, Italy; 3 Department of Medicine and Surgery, University of Milano Bicocca, Monza, Italy; 4 Department of Mental Health, ASST Ovest Milanese, Milano, Italy; 5 Unit of Epidemiological and Evaluation Psychiatry, IRCCS Istituto Centro San Giovanni di Dio Fatebenefratelli, Brescia, Italy; 6 Division of Psychiatry, University College London, London, UK; 7 Department of Psychology, Catholic University of the Sacred Heart, Milano, Italy; 8 Department of Mental Health, ASST of Monza, Monza, Italy; 9 Department of Mental Health, ASST Spedali Civili of Brescia, Brescia, Italy; 10 Department of Mental Health, Asst-Rhodense G.Salvini of Garbagnate, Milano, Italy

Correction to: *Scientific Reports* 10.1038/s41598-019-53993-7, published online 28 November 2019

The original version of this Article contained errors. In the title of the paper, the word “in” was incorrectly given as “In”.

In the original Table 1, cells in column six were not merged correctly and the table has been replaced.

Finally, the original Figures 1 and 2 have been replaced with higher resolution images.

These errors have now been corrected in the PDF and HTML versions of the Article. The accompanying Supplementary Information file was correct at the time of publication.

