# Supplementary material for: Self-harm behaviour and externally-directed aggression in psychiatric outpatients: a multicentre, prospective study (viormed-2 study)
Source: Sci Rep. 2019 Nov 28;9:17857. doi: 10.1038/s41598-019-53993-7 (PMC6882905; doi:10.1038/s41598-019-53993-7)
Supplement: Supplementary file 1 — Supplementary information [file 41598_2019_53993_MOESM1_ESM.pdf]

Paolo Scocco[1], Ambra Macis[2], Clarissa Ferrari[2], Mattia Bava[3], Giorgio Bianconi[4], Viola Bulgari[5], Valentina Candini[5], Giuseppe Carrà[3,6], Cesare Cavalera[5,7], Massimo Clerici[3,8], Giovanni Conte[9], Marta Cricelli[10], Maria Teresa Ferla[10], Laura Iozzino[5], Alberto Stefana[9], Giovanni de Girolamo[5]

SELF-HARM BEHAVIOUR AND EXTERNALLY-DIRECTED AGGRESSION IN PSYCHIATRIC OUTPATIENTS: A MULTICENTRE, PROSPECTIVE STUDY (VIORMED-2 STUDY)

SUPPLEMENTARY FILES

Table 1S
Socio-demographic characteristics of the sample

	V<br>N=73<br>n (%)	V-SH<br>N=53<br>n (%)	SH<br>N=34<br>n (%)	CONT<br>N=86<br>n (%)	*p-value*
**Age Mean (SD)**	45.4 (10.2)	44.2 (9.8)	46.5 (12.0)	43.8 (10.9)	0.552
**Sex**					
Male	63 (86.3)	40 (75.5)	20 (58.8)	69 (80.2)	**0.014**
Female	10 (13.7)	13 (24.5)	14 (41.2)	17 (19.8)	
**Nationality**					
Italian	71 (97.3)	50 (94.3)	33 (97.1)	85 (98.8)	0.439 #
Other	2 (2.7)	3 (5.7)	1 (2.9)	1 (1.2)	
**Marital status**					
Married or cohabitant	27 (37.0)	24 (45.3)	17 (50.0)	30 (34.9)	0.355
Single	46 (63.0)	29 (54.7)	17 (50.0)	56 (65.1)	
**Education**					
Low level	48 (65.8)	34 (64.2)	15 (44.1)	48 (55.8)	0.143
Medium-high level	25 (34.2)	19 (35.8)	19 (55.9)	38 (44.2)	
**Occupation**					
Employed	29 (39.7)	23 (44.2)	12 (36.4)	48 (56.5)	0.102
Unemployed	44 (60.3)	29 (55.8)	21 (63.6)	37 (43.5)	
**Social support in the last year**					
Present	61 (85.9)	41 (80.4)	26 (78.8)	72 (84.7)	0.738
Absent	10 (14.1)	10 (19.6)	7 (21.2)	13 (15.3)	
**Time spent doing nothing**					
Less than 3 h/day	28 (38.9)	18 (35.3)	16 (48.5)	49 (57.6)	**0.036**
More than 3 h/day	44 (61.1)	33 (64.7)	17 (51.5)	36 (42.4)	
**Episodes of violence in family**					
Yes	13 (19.4)	27 (54.0)	7 (21.2)	4 (4.9)	**<0.001**
No	54 (80.6)	23 (46.0)	26 (78.7)	77 (95.1)	

V: patients with a history of violent behaviour against other people (but not self-harm behaviour); V-SH: patients with both a history of violent behaviour against other people and self-harm behaviour; SH: patients with a history of self-harm behaviour; CONT: patients with no history of violent behaviour against other people and of self-harm behaviour.

For continuous variables Mean (SD) is reported and corresponding p-value was evaluated through ANOVA and Kruskal-Wallis test. For categorical variables frequency (%) is reported and corresponding p-value was obtained through χ^2 test or Fisher's exact test (when n<5 at least in one cell).

Table 2S
Clinical rating scale scores at baseline by groups

	**V (1) N=73 M(SD)**	**V-SH (2) N=53 M(SD)**	**SH (3) N=34 M(SD)**	**CONT (4) N=86 M(SD)**	*p-value*	Post-hoc
**BGLHA**						
Total score	36.8 (10.4)	44.8 (13.5)	37.8 (11.0)	32.2 (8.8)	**<0.001**	(2) vs (4) <0.001
**BPRS**						
Affect-Anxiety	10.7 (4.0)	12.2 (4.2)	11.1 (4.2)	10.9 (4.4)	0.136	
Activation	12.2 (5.2)	11.8 (5.2)	9.9 (3.1)	9.5 (3.0)	**0.001**	**(1) vs (4) 0.002**<br>**(2) vs (4) 0.038**
Negative Symptoms	5.7 (3.3)	4.9 (2.4)	5.5 (2.8)	4.8 (3.0)	0.696	
Psychotic Symptoms	12.8 (5.9)	11.1 (4.1)	10.8 (2.7)	11.1 (3.8)	0.207	
Depression (item 3)	1.7 (1.0)	2.2 (1.2)	2.0 (1.2)	1.7 (1.0)	**0.040**	**(1) vs (2) <0.1**
Total Score	41.3 (12.3)	40.6 (11.0)	37.3 (8.6)	36.7 (9.1)	0.115	
**SLOF**						
Physical functioning	24.1 (1.4)	24.2 (1.5)	24.3 (0.9)	24.2 (1.5)	0.896	
Self-care	33.3 (2.9)	33.2 (2.9)	33.7 (2.8)	33.3 (3.2)	0.883	
Interpersonal relationships	23.4 (6.0)	24.6 (5.9)	26.1 (6.1)	24.4 (5.4)	0.173	
Social acceptability/adjustment	24.5 (3.4)	22.5 (4.6)	26.8 (2.9)	27.1 (2.6)	**<0.001**	(1) vs (2) 0.009<br>(1) vs (3) 0.009<br>(1) vs (4) <0.001<br>(2) vs (3) < 0.001<br>(2) vs (4) <0.001
Activities	48.0 (7.7)	49.3 (6.9)	50.4 (5.1)	49.6 (6.4)	0.292	
Work skills	21.4 (6.8)	21.7 (6.4)	22.7 (6.5)	23.5 (5.9)	0.171	
**BDHI**						
Assault	4.0 (2.6)	5.4 (2.5)	4.0 (2.3)	4.6 (2.5)	**0.046**	(1) vs (2) 0.058
Indirect aggression	4.0 (2.2)	5.5 (2.1)	5.4 (1.3)	4.2 (1.8)	**<0.001**	(1) vs (2) <0.001<br>(1) vs (3) 0.008<br>(2) vs (4) 0.008<br>(3) vs (4) 0.034
Verbal aggression	6.0 (2.7)	7.3 (2.9)	6.5 (2.6)	6.2 (2.5)	0.099	
Irritability	3.8 (2.6)	4.6 (2.6)	4.6 (2.5)	3.8 (2.2)	0.149	
Negativism	2.3 (1.5)	3.0 (1.6)	2.2 (1.6)	2.4 (1.6)	0.096	
Resentment	3.7 (1.9)	4.2 (2.1)	4.7 (2.2)	3.6 (2.3)	0.143	
Suspicion	4.0 (2.6)	4.3 (2.1)	4.8 (2.7)	4.1 (2.6)	0.560	
Guilt	4.9 (2.3)	4.9 (2.4)	5.3 (2. 3)	4.0 (2.2)	0.055	
Total score	33.0 (14.7)	38.8 (14.0)	36.9 (12.5)	34.1 (12.4)	0.263	
**BIS**						
Attentional impulsiveness	15.0 (3.9)	16.0 (4.8)	15.3 (4.1)	14.2 (3.7)	0.118	
Motor impulsiveness	22.4 (4.9)	23.0 (4.8)	22.9 (5.4)	20.8 (4.1)	**0.028**	(2) vs (4) < 0.1
Non-planning impulsiveness	27.4 (5.0)	27.0 (5.9)	27.6 (6.2)	25.9 (5.0)	0.278	
Total score	64.5 (10.9)	65.3 (12.6)	65.8 (11.9)	60.6 (9.4)	**0.035**	------
**STAXI -2**						
State anger	33.3 (17.2)	33.7 (24.1)	29.8 (17.6)	32.3 (17.8)	0.713	
Feeling angry	25.3 (21.3)	23.3 (27.1)	21.9 (20.8)	27.4 (24.3)	0.587	
Feel like expressing anger verbally	25.7 (22.8)	22.9 (26.8)	21.5 (21.9)	25.8 (21.9)	0.546	
Feel like expressing anger physically	24.5 (22.3)	21.6 (25.2)	22.1 (23.9)	24.6 (21.8)	0.754	
Trait anger	33.1 (19.9)	32.8 (18.9)	29.5 (16.4)	29.1 (15.9)	0.683	
Angry temperament	27.6 (24.6)	22.3 (22.5)	22.9 (22.1)	24.5 (20.9)	0.594	
Angry reaction	26.7 (23.5)	23.5 (23.3)	22.0 (19.6)	23.8 (19.1)	0.941	
Anger expression-out	30.5 (21.1)	29.5 (21.0)	25.3 (16.3)	26.7 (16.5)	0.390	
Anger expression-in	32.0 (20.7)	29.4 (19.0)	31.0 (19.4)	32.3 (19.1)	0.990	
Anger control-out	29.5 (13.3)	25.6 (13.9)	28.4 (15.1)	35.1 (15.2)	**0.001**	(2) vs (4) <0.001

Anger control-in	32.1 (15.2)	29.8 (16.0)	33.1 (17.5)	36.1 (16.3)	0.070	
Anger expression Index	45.5 (16.6)	47.8 (17.3)	42.8 (15.7)	38.8 (14.9)	**0.020**	(2) vs (4) 0.027

V: patients with a history of violent behaviour against other people (but not self-harm behaviour); V-SH: patients with both a history of violent behaviour against other people and self-harm behaviour; SH: patients with a history of self-harm behaviour; CONT: patients with no history of violent behaviour against other people and of self-harm behaviour.

p-value was evaluated through ANOVA or Kruskal-Wallis test.
------ From post-hoc analysis did not emerge any statistically significant difference

Table 3S
Personality assessment with MCMI-III at baseline by groups

**MCMI-III**	**V N=73 n (%)**	**V-SH N=53 n (%)**	**SH N=34 n (%)**	**CONT N=86 n (%)**	*p-value*
**Schizoid**					
No	44 (62.9)	33 (66.0)	23 (69.7)	52 (62.7)	
Maladaptive trait	8 (11.4)	4 (8.0)	2 (6.1)	5 (6.0)	0.898
Disorder	18 (25.7)	13 (26.0)	8 (24.2)	26 (31.3)	
**Avoidant**					
No	39 (55.7)	27 (54.0)	17 (51.5)	40 (48.2)	
Maladaptive trait	13 (18.6)	5 (10.0)	8 (24.2)	14 (16.9)	0.548
Disorder	18 (25.7)	18 (36.0)	8 (24.2)	29 (34.9)	
**Depressive**					
No	41 (58.6)	26 (52.0)	18 (54.6)	50 (60.2)	
Maladaptive trait	11 (15.7)	5 (10.0)	4 (12.1)	10 (12.0)	0.818
Disorder	18 (25.7)	19 (38.0)	11 (33.3)	23 (27.7)	
**Dependent**					
No	36 (51.4)	28 (56.0)	21 (63.6)	145 (54.2)	
Maladaptive trait	13 (18.6)	8 (16.0)	3 (9.1)	6 (7.2)	0.341
Disorder	21 (30.0)	14 (28.0)	9 (27.3)	32 (38.6)	
**Histrionic**					
No	49 (70.0)	31 (62.0)	23 (69.7)	52 (62.7)	
Maladaptive trait	8 (11.4)	7 (14.0)	3 (9.1)	8 (9.6)	0.857
Disorder	13 (18.6)	12 (24.0)	7 (21.2)	23 (27.7)	
**Narcissistic**					
No	41 (58.6)	31 (62.0)	28 (84.8)	54 (65.1)	
Maladaptive trait	10 (14.3)	6 (12.0)	1 (3.0)	8 (9.6)	0.268
Disorder	19 (27.1)	13 (26.0)	4 (12.1)	21 (25.3)	
**Antisocial**					
No	53 (75.7)	36 (72.0)	27 (81.8)	61 (73.5)	
Maladaptive trait	2 (2.9)	2 (4.0)	1 (3.0)	5 (6.0)	0.937
Disorder	15 (21.4)	12 (24.0)	5 (15.2)	17 (20.5)	
**Sadistic**					
No	59 (84.3)	38 (76.0)	27 (81.8)	65 (78.3)	
Maladaptive trait	0 (0.0)	0 (0.0)	0 (0.0)	0 (0.0)	°
Disorder	11 (15.7)	12 (24.0)	6 (18.2)	18 (21.7)	
**Compulsive**					
No	34 (48.6)	25 (50.0)	19 (57.6)	30 (36.1)	
Maladaptive trait	4 (5.7)	1 (2.0)	1 (3.0)	7 (8.4)	0.319
Disorder	32 (45.7)	24 (48.0)	13 (39.4)	46 (55.4)	
**Negativistic**					
No	36 (51.4)	21 (42.0)	23 (69.7)	47 (56.6)	
Maladaptive trait	13 (18.6)	12 (24.0)	4 (12.1)	12 (14.5)	0.313
Disorder	21 (30.0)	17 (34.0)	6 (18.2)	24 (28.9)	
**Masochistic**					
No	57 (81.4)	40 (80.0)	26 (78.8)	64 (77.1)	
Maladaptive trait	0 (0.0)	0 (0.0)	0 (0.0)	0 (0.0)	°
Disorder	13 (18.6)	10 (20.0)	7 (21.2)	19 (22.9)	

**Schizotypal**					
No	53 (75.7)	33 (66.0)	23 (69.7)	59 (71.1)	
Maladaptive trait	2 (2.9)	4 (8.0)	3 (9.1)	4 (4.8)	0.773
Disorder	15 (21.4)	13 (26.0)	7 (21.2)	20 (24.1)	
**Borderline**					
No	40 (57.1)	19 (38.0)	19 (57.6)	50 (60.2)	
Maladaptive trait	9 (12.9)	4 (8.0)	5 (15.2)	11 (13.3)	0.066
Disorder	21 (30.0)	27 (54.0)	9 (27.3)	22 (26.5)	
**Paranoid**					
No	46 (65.7)	29 (58.0)	25 (75.8)	58 (69.9)	
Maladaptive trait	4 (5.7)	0 (0.0)	1 (3.0)	3 (3.6)	0.274
Disorder	20 (28.6)	21 (42.0)	7 (21.2)	22 (26.5)	

V: patients with a history of violent behaviour against other people (but not self-harm behaviour); V-SH: patients with both a history of violent behaviour against other people and self-harm behaviour; SH: patients with a history of self-harm behaviour; CONT: patients with no history of violent behaviour against other people and of self-harm behaviour.

p-value was obtained through χ^2 test or Fisher's exact test (when n<5 at least in one cell).
° The p-value could not be estimated.

Table 4S

Predictors of aggressive and violent behavior: generalized linear models (GLMs) with interaction effect between variables and groups (all sample), and corresponding GLMs for the four groups

Dependent: MOAS Total score					
		Groups			
	All sample	V	V-SH	SH	CONT
**Independent variables**	**pvalue°**	β_V	$\beta_{V\text{-}SH}$	β_{SH}	β_{CONT}
**MCMI Negativistic**<br>(2=Dis; 1=Trait; 0=No-ref)	<0.001	2 vs 0 2.10<br>1 vs 0 1.00	2 vs 0 1.56<br>1 vs 0 0.51	**2 vs 0 2.62***<br>**1 vs 0 8.38***	**2 vs 0 1.83****<br>**1 vs 0 5.20****
**BIS Total Score**	<0.001	1.01	1.02	**1.08****	**0.95****
**Lifetime Substances use**<br>(Yes vs No-ref)	<0.001	1.09	1.08	**4.44****	**5.98****
**BIS Motor Impulsiveness**	0.001	1.01	**1.08***	**1.22****	**0.91***
**Interpersonal relationships**<br>(Yes vs No-ref)	0.001	0.42*	1.77	0.40	**8.18****
**SLOF Social acceptability**	0,001	0.94	**0.90****	**0.80***	**0.73****
**MCMI Borderline**<br>(2=Dis; 1=Trait; 0=No-ref)	0.002	2 vs 0 2.29*<br>1 vs 0 1.01*	2 vs 0 1.43<br>1 vs 0 1.70	**2 vs 0 4.63****<br>**1 vs 0 12.07****	2 vs 0 0.40<br>1 vs 0 1.21
**BIS Non Planning Impulsiveness**	0,002	0.99	1.02	**1.10***	**0.90****
**BGLHA**	0.002	1.04*	0.99	**1.13****	1.01
**Time spent doing nothing** (<3 vs >3-ref)	0.003	0.41**	0.69	0.70	**2.50****
**MCMI Schizotypal**<br>(2=Dis; 1=Trait; 0=No-ref)	0.008	2 vs 0 1.50*<br>1 vs 0 0.20*	**2 vs 0 2.27***<br>**1 vs 0 3.07***	2 vs 0 1.00<br>1 vs 0 0.09	2 vs 0 0.79<br>1 vs 0 0.18
**BPRS Activation**	0.008	1.05	**1.06***	**1.26****	**1.20****
**MCMI Istrionica**<br>(2=Dis; 1=Trait; 0=No-ref)	0.014	**2 vs 0 2.10****<br>**1 vs 0 0.99****	2 vs 0 1.95<br>1 vs 0 1.26	2 vs 0 0.96<br>1 vs 0 0.41	2 vs 0 0.51<br>1 vs 0 1.40
**STAXI Index**	0.022	1.01	**1.04****	**1.07****	1.02
**BIS Attentional Impulsiveness**	0.035	1.05	1.02	**1.16***	0.94
**BDHI Indirect aggression**	0.050	0.93	1.07	**1.61***	0.94
**Dependent: MOAS Verbal Aggression**					
**BIS Motor Impulsiveness**	0.001	1.02	1.07	**1.25****	0.94
**Interpersonal relationships**<br>(Yes vs No-ref)	0.002	**0.38****	1.54	0.53	**5.14****
**BIS Total Score**	0.002	1.01	1.03	**1.07****	**0.97***
**Lifetime Substances use** (Si vs No-ref)	0.004	1.24	0.96	**3.62***	**4.49****
**BPRS Total Score**	0.015	1.00	**1.04****	1.04	**1.06****
**MCMI Negativistic**<br>(2=Dis; 1=Trait; 0=No-ref)	0.016	2 vs 0 2.11<br>1 vs 0 1.36	2 vs 0 1.53<br>1 vs 0 0.66	**2 vs 0 3.38***<br>**1 vs 0 6.36***	**2 vs 0 1.27****<br>**1 vs 0 3.74****
**SLOF Social acceptability**	0.018	**0.91***	**0.89****	**0.83***	**0.75****
**STAXI Index**	0.022	1.01	**1.04****	**1.07****	1.02
**BGLHA**	0.024	1.02	0.99	**1.11***	1.00
**MCMI Borderline**<br>(2=Dis; 1=Trait; 0=No-ref)	0.036	**2 vs 0 2.45***<br>**1 vs 0 1.77***	2 vs 0 1.32<br>1 vs 0 1.76	**2 vs 0 4.19***<br>**1 vs 0 6.53***	2 vs 0 0.53<br>1 vs 0 1.32
**BIS Non Planning Impulsiveness**	0.045	1.01	1.05	1.06	**0.94***
**Dependent: MOAS Aggression against Objects**					

**BIS Total Score**	0.004	1.02	1.01	**1.12****	0.93
**Time spent doing nothing** (<3 vs >3-ref)	0.007	0.44	0.83	0.72	**16.06***
**BIS Motor Impulsiveness**	0.009	1.03	1.04	**1.33****	0.80
**Lifetime Substances use** (Yes vs No-ref)	0.010	0.50	0.96	**5.62***	**6.96***
**BIS Non Planning Impulsiveness**	0.022	1.01	0.99	1.11	**0.84***
**MCMI Negativistic** (2=Dis; 1=Trait; 0=No-ref)	0.031	**2 vs 0 4.00*** **1 vs 0 1.66***	2 vs 0 0.93 1 vs 0 0.40	2 vs 0 4.89 1 vs 0 6.11	**2 vs 0 3.50*** **1 vs 0 14.96***

V: patients with a history of violent behaviour against other people (but not self-harm behaviour); V-SH: patients with both a history of violent behaviour against other people and self-harm behaviour; SH: patients with a history of self-harm behaviour; CONT: patients with no history of violent behaviour against other people and of self-harm behaviour.

ref indicates the baseline category
p value: significance of the interaction term between the group variable and the independent variables
* $p<0.05$; ** $p<0.01$
β: exponential transformation of the coefficients of the Generalized Linear Models.
Bv, $\beta_{V\text{-}SH}$, β_{SH}, β_{CONT}: estimates of the variable effect in the four groups separately
